# The Utility of Metabolic Parameters on Baseline F-18 FDG PET/CT in Predicting Treatment Response and Survival in Paediatric and Adolescent Hodgkin Lymphoma

**DOI:** 10.3390/jcm10245979

**Published:** 2021-12-20

**Authors:** Janet Denise Reed, Andries Masenge, Ane Buchner, Fareed Omar, David Reynders, Mariza Vorster, Christophe Van de Wiele, Mike Sathekge

**Affiliations:** 1 Department of Nuclear Medicine, University of Pretoria, Pretoria 0002, South Africa; marizavorster@gmail.com; 2 Steve Biko Academic Hospital, Pretoria 0002, South Africa; ane.buchner@up.ac.za (A.B.); fareed.omar@up.ac.za (F.O.); david.reynders@up.ac.za (D.R.); 3 Department of Statistics, University of Pretoria, Pretoria 0002, South Africa; andries.masenge@up.ac.za; 4 Department of Paediatric Oncology, University of Pretoria, Pretoria 0002, South Africa; 5 Department of Radiology and Nuclear Medicine, University of Ghent, 9000 Ghent, Belgium

**Keywords:** F-18 FDG PET/CT (fluoro-deoxy-glucose positron emission tomography/computed tomography), metabolic parameters, total metabolic tumor volume (tMTV), total lesion glycolysis (TLG), maximum standard uptake value (SUVmax), paediatric oncology, Hodgkin lymphoma (HL), overall survival (OS), progression-free survival (PFS), human immunodeficiency virus (HIV)

## Abstract

Lymphoma is the third most common paediatric cancer. Early detection of high-risk patients is necessary to anticipate those who require intensive therapy and follow-up. Current literature shows that residual tumor avidity on PET (Positron Emission Tomography) following chemotherapy corresponds with decreased survival. However, the value of metabolic parameters has not been adequately investigated. In this retrospective study, we aimed to evaluate the prognostic value of metabolic and other parameters in paediatric and adolescent Hodgkin lymphoma. We recorded tMTV (total Metabolic Tumor Volume), TLG (Total Lesion Glycolysis), and SUVmax (maximum Standard Uptake Value) on baseline PET, as well the presence of bone marrow or visceral involvement. HIV (human immunodeficiency virus) status and baseline biochemistry from clinical records were noted. All patients received stage-specific standard of care therapy. Response assessment on end-of-treatment PET was evaluated according to the Deauville criteria. We found that bone marrow involvement (*p* = 0.028), effusion (*p* < 0.001), and treatment response (*p* < 0.001) on baseline PET, as well as HIV status (*p* = 0.036) and baseline haemoglobin (*p* = 0.039), were significantly related to progression-free survival (PFS), whereas only effusion (*p* = 0.017) and treatment response (*p* = 0.050) were predictive of overall survival (OS). Only baseline tMTV predicted treatment response (*p* = 0.017). This confirms the value of F-18 FDG PET/CT (Fluoro-deoxy-glucose Positron Emission Tomography/Computed Tomography) in prognostication in paediatric and adolescent Hodgkin lymphoma; however, further studies are required to define the significance of metabolic parameters.

## 1. Introduction

Lymphoma is the third most common paediatric malignancy, superseded only by leukemia and central nervous system tumors [1,2]. Collectively, they account for approximately 12% of all childhood cancers, of which about 40% are represented by Hodgkin lymphoma (HL), while the remaining 60% comprise a diverse group of lymphoid malignancies, the non-Hodgkin lymphomas (NHL) [2,3,4]. Unlike lymphoma in adults, indolent lymphomas are rare in children [4]. Despite the aggressive nature of most paediatric lymphomas, advances in management with a combination of chemotherapy and targeted radiation therapy have resulted in favorable outcomes, with 5-year survival rates reported at 95% and 78% for HL and NHL, respectively [2]. In view of these improvements in survival, current interest has shifted to reducing treatment-related sequelae, which include secondary malignancies and cardiovascular events [1,3,4]. Early identification of patients at risk of treatment failure (poor response to first-line therapy or initial response with subsequent relapse) should be prioritized to improve survival while minimizing these treatment-related adverse effects [2]. Current risk-stratification criteria fail to identify these children. Another consideration in the context of a resource-limited developing country is the additional burden of diseases such as the human immunodeficiency virus (HIV), which may result in delayed and altered clinical presentation and poor treatment response [5].

There is a clear need to accurately and promptly risk-stratify paediatric patients, to implement risk-adapted treatment strategies. F-18 FDG PET/CT has been established as a powerful tool for both initial staging and early response assessment in HL, allowing patient management to be individualized. Current literature shows that residual tumor avidity on PET following chemotherapy corresponds with lower overall survival (OS) and progression-free survival (PFS), whereas the opposite is true in the case of a negative PET following therapy [3,4]. However, there is a paucity of data regarding the value of various metabolic parameters (namely whole-body metabolic tumor volume, [WB-MTV or tMTV] and total lesion glycolysis [TLG]) on baseline F-18 FDG PET/CT for risk-stratification and prediction of OS and PFS. The most well-studied metabolic parameter, the maximum standard uptake value (SUV_max_), is not always reproducible as its measurement is influenced by a number of different factors, and it is therefore not always reliable for prognostication [2,6,7,8,9]. Metabolic volumetric parameters, on the other hand, give an indication of the whole-body tumor burden, and have recently been proposed as a valuable tool for prognostication. However, data are limited (especially in paediatric cohorts) and somewhat conflicting; therefore, there is a need to further investigate these promising parameters, which have the potential to significantly impact the management of paediatric lymphoma by proper risk-stratification, potentially predicting treatment response and survival [2,10,11,12]. The ability to identify high-risk patients who will require more intensive therapy and follow-up at the outset will ultimately improve patient outcomes. Also of importance is the de-escalation of therapy when complete metabolic response is achieved, to reduce late effects of treatment in children who have an otherwise excellent prognosis [1,3,4].

The aim of this retrospective study was to evaluate the prognostic value of tMTV and TLG (compared with SUV_max_) on baseline F-18 FDG PET/CT in predicting metabolic response to treatment, overall survival, and progression-free survival in paediatric and adolescent Hodgkin lymphoma patients. Secondary objectives were to compare the performance of these parameters to other imaging and biochemical risk factors in prognostication, and to evaluate the value of initial treatment response as a predictor of PFS and OS (as determined by the Deauville scoring system).

## 2. Materials and Methods

### 2.1. Patients

All paediatric and adolescent patients (≤20 years) with histologically confirmed Hodgkin lymphoma who were referred to the Department of Nuclear Medicine, Steve Biko Academic Hospital for baseline F-18 FDG PET/CT imaging between January 2008 and December 2020 were considered for inclusion in this retrospective study. Patients were excluded if (i) any lymphoma-related treatment (chemotherapy, radiation therapy, surgical intervention other than biopsy) was received prior to baseline F-18 FDG PET/CT imaging; (ii) F-18 FDG PET/CT images were sub-optimal or the acquisition protocol deviated from the standard; (iii) no pathological uptake was noted on baseline imaging; (iv) therapy was defaulted or not in accordance with standard regimes; or (v) no end-of-treatment (EOT) F-18 FDG PET/CT was performed or the patient was lost to follow-up (<5 years of clinical follow-up).

All patients were treated according to the standard of care treatment for their specific disease stage by a specialist paediatric oncologist. End-of-treatment (EOT) F-18 FDG PET/CT was obtained ≥2 weeks after completion of first-line therapy (6 cycles of ABVD—Adriamycin, Bleomycin, Vincristine, Dacarbazine).

Characteristics of enrolled patients were recorded, including epidemiological features (age at diagnosis and gender), histological sub-type, stage (early [I and II] versus advanced [III and IV] disease), presence of B-symptoms, HIV status, and baseline biochemistry (haemoglobin [Hb], albumin, and lacatate dehydrogenase [LDH]). Image-derived parameters (such as the presence or absence of bone marrow; splenic, liver, or lung involvement; the presence of effusions; and/or the presence of bulky disease) were recorded on baseline and EOT F-18 FDG PET/CT scans, as detailed below. 

This retrospective study was approved by the Research Ethics Committee, University of Pretoria, and was carried out in accordance with the Declaration of Helsinki.

### 2.2. F-18 FDG PET/CT Image Acquisition

F-18 FDG PET/CT image acquisition (for both baseline and EOT scans) strictly followed the departmental protocol, which is in accordance with international guidelines. All patients fasted for a minimum of 6 h prior to imaging, and blood glucose levels were ≤11 mmol/L in all cases. The dose of F-18 FDG was calculated at 3 MBq/kg body weight (minimum dose of 14 MBq), as per EANM guidelines. Image acquisition was commenced at 60 +/− 5 min post-tracer injection using a Biograph 40 Truepoint PET/CT scanner (Siemens Medical Solution, IL, USA). Unless contraindicated, both oral and intravenous contrast were administered, adjusted for the patient’s weight. For oral contrast, 6–10 mL of Omnipaque 300 (Iohexol 300mg iodine/mL, GE Healthcare Pty Ltd, Midrand, South Africa.) was mixed with 300–500 mL of water and administered more than 1 h prior to imaging; alternatively, water was administered. For intravenous contrast, 2 mL/kg of Omnipaque 300 or Jopamiron 300 (Iopamidol 300mg iodine/mL, Africa X-Ray Industrial and Medical, Midrand, South Africa) intravenous contrast was provided, with a scan delay time of 60–80 s. The CT parameters for children <13 years were as follows: 120 KeV, 60 mAs, with a section width of 3 mm and a pitch of 0.8. The adult CT parameters were applied for children and adolescents ≥13 years: 120 KeV, 150, with a section width of 5 mm and a pitch of 0.8. PET imaging was acquired in 3D mode at 3 min per bed position, from vertex to mid-thigh. The CT data were used for attenuation correction. Image reconstruction was done with the ordered subset expectation maximization iterative reconstruction algorithm (4 iterations, 8 subsets).

### 2.3. Image Analysis

The reconstructed images were displayed on a dedicated workstation equipped with syngo software (Siemens Medical Solutions, IL, USA). Image interpretation was performed by a qualified nuclear medicine physician with a minimum of 5 years of experience and a senior nuclear medicine registrar.

#### 2.3.1. Measurement of Quantitative PET-Derived Parameters

For the determination of MTV, a semi-automated spherical volume of interest was drawn around each malignant lesion. Each VOI was adjusted manually to exclude areas of intense physiological uptake contiguous to the lesion (e.g., bladder, myocardium, and thymus). An SUV threshold of 2.5 and a 3D isocontour of 41% was applied. The sum of MTV measurements for all lesions was manually calculated to determine the whole-body MTV (tMTV).

TLG was calculated for each lesion by multiplying its MTV by the SUV_mean_. The sum of the TLG measurements for all lesions was manually calculated to determine the whole-body TLG.

In addition, the SUVmax of the most intense lesion was recorded.

The abovementioned specifications were used for both baseline and EOT F-18 FDG PET/CT scans.

#### 2.3.2. Identification Criteria for Bone Marrow, Splenic and Liver Involvement, and Bulky Disease

Bone marrow involvement was considered positive in the presence of focal or multifocal uptake. Generalized marrow activation with an underlying explanation, e.g., documented anaemia, was not considered positive for involvement. Bone marrow biopsy results, when performed, assisted in determining involvement in unclear cases.

Splenic involvement was noted if there was diffuse uptake, a solitary mass, miliary lesions, nodules on PET/CT, or >13 cm in length on CT, as per the Lugano criteria [13].

Similarly, liver involvement was noted by the presence of diffusely increased uptake, a mass, or nodules.

The presence of pleural and pericardial effusions was noted, as well as disease bulk as defined by the Lugano criteria, i.e.,, a single nodal mass of ≥10 cm or ≥1/3 of the trans-thoracic diameter at any level of the thoracic vertebrae, as determined by CT [13].

#### 2.3.3. Response Evaluation

The metabolic response to treatment was determined using the Deauville visual scoring system after first-line therapy in all patients. A score of 1–3 was considered PET negative- complete metabolic response (CMR). A score of 4–5 was considered PET positive: partial metabolic response (PMR) if there was a decrease in uptake from baseline; progressive metabolic disease (PMD) if there was an increase in uptake or if new lesions were noted; no metabolic response (NMR) if there was no significant change in uptake. In patients for whom no EOT PET was available, CR (complete response, surrogate to CMR) was assumed on clinical grounds if the patient was followed up for ≥5 years with no evidence of progression, i.e., complete remission.

In situations where second-line therapy was instituted or consolidation radiation therapy administered with multiple subsequent PET scans, only the first end-of-therapy (but not interim) scan was considered for evaluation for standardization purposes.

### 2.4. Clinical Data and Follow-up

Various clinical and biochemical parameters were recorded at baseline as follows: presence of B-symptoms; HIV status; baseline Hb level (considered low if <10.5 g/dL); LDH level (considered elevated if >2 × the upper limit of normal [ULN]); and serum albumin level (considered low if <35 g/L). B-symptoms were defined by the presence of fever (temperature > 38 °C), drenching night sweats, and unexplained loss of >10% of body weight within the preceding 6 months

In addition to follow-up F-18 FDG PET/CT imaging, patients were followed up clinically. Progression-free survival (PFS) was determined from the date of baseline F-18 FDG PET/CT imaging (surrogate for the time of commencing treatment) to the time of first disease progression/relapse, death, or last follow-up date. PFS was inclusive of all disease events. Disease progression was determined on clinical, biochemical, and imaging grounds. Disease progression on PET was defined by Deauville 4 or 5, with an increase in uptake from baseline and/or if new lesions were noted. 

Overall survival (OS) was determined from the date of baseline F-18 FDG PET/CT imaging to the time of death or last follow-up. OS was inclusive of all deaths.

### 2.5. Statistical Analysis

Statistical analysis was performed using the commercially available software package SPSS, version 28.0 (IBM SPSS, Ghent, Belgium). The Kolmogorov-Smirnov test was used to determine if data were normally distributed. Quantitative variables were compared using a paired Student’s *t*-test and ANOVA when normally distributed, or a Mann-Whitney test and a Kruskal-Wallis test when not normally distributed. ROC curve analysis was performed for clinically relevant parameters.

For univariate and regression analysis, we dichotomized values according to the median values in case of continuous variables. We also dichotomized the following clinical covariates: age; gender; disease stage (stage I + II and stage III + IV); the presence of B-symptoms; HIV status; Hb, albumin and LDH values; the presence of lesions in the bone marrow, spleen, liver, or lungs; the presence of pleural or pericardial effusion; the presence of bulky disease; and treatment response (CMR versus PMR + PMD). PFS and OS were estimated by the Kaplan-Meier method and log-rank testing to examine the predictive value of dichotomized variables and other clinical risk factors for disease control and OS. Multivariate analysis was performed using Cox-regression and included in the sequential order of statistical significance variables that were found to be significant in the univariate analysis, followed by the interactive terms.

Finally, the Chi-square test was used to determine differences in proportion as appropriate.

## 3. Results

### 3.1. Patient Characteristics

Patient characteristics are presented in Table 1.

A total of 69 patients, 33 females and 36 males, were included in this study. The median age of the patient population under study at the time of data analysis was 11 years (range 4–20 years). Age proved not to be significantly different between females and males (*p* = 0.46). One patient presented with stage I disease, 25 patients suffered from stage II disease, 17 patients suffered from stage III disease, and 26 patients suffered from stage IV disease. EOT F-18 FDG PET/CT was obtained in 59 of the 69 patients at a mean time of 7.31 weeks post-completion of first-line chemotherapy (range: 2–17 weeks). Response rates to the initiated treatment according to the Lugano-criteria were as follows: there were 40 complete responders (CR) (37 CMR via EOT PET and three clinical CR determined by ≥5-year progression-free clinical follow-up), 18 partial metabolic responders, four patients who progressed under treatment (progressive metabolic disease), and seven patients who died before EOT PET. The mean clinical follow-up period was 50.48 months.

### 3.2. FDG PET CT Imaging

**Figure 1:**
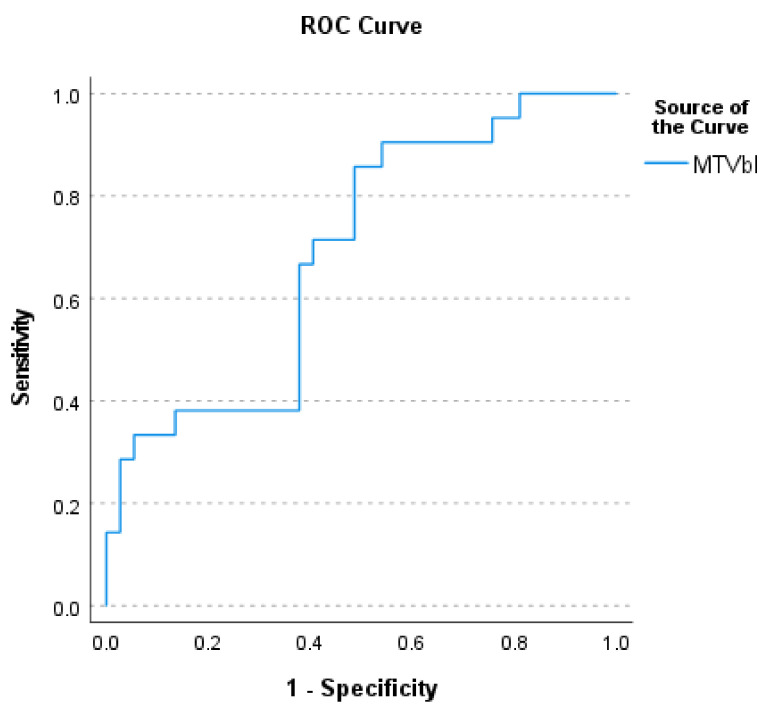
ROC curve showing the diagnostic ability of MTVbl (metabolic tumor volume derived from the baseline FDG-PET/CTscan) to discriminate complete responders from partial and non-responders.

The median SUVmax value of the most active lesions in all patients was 11.7 (range: 3.5–29.7). The median MTV and TLG of all summed metabolic active lymphoma lesions were, respectively, 273.9 mL (range 1.1–1986.7 mL) and 1484.6 mlxSUV (range: 3.0–14,048.28 mlxSUV). Baseline MTV values of patients with complete metabolic results proved to be significantly lower than the values of patients with a partial metabolic response or a progressive metabolic disease (*p* = 0.017, ROC-AUC = 0.690) (see Figure 1).

### 3.3. Progression-Free Survival

**Figure 2:**
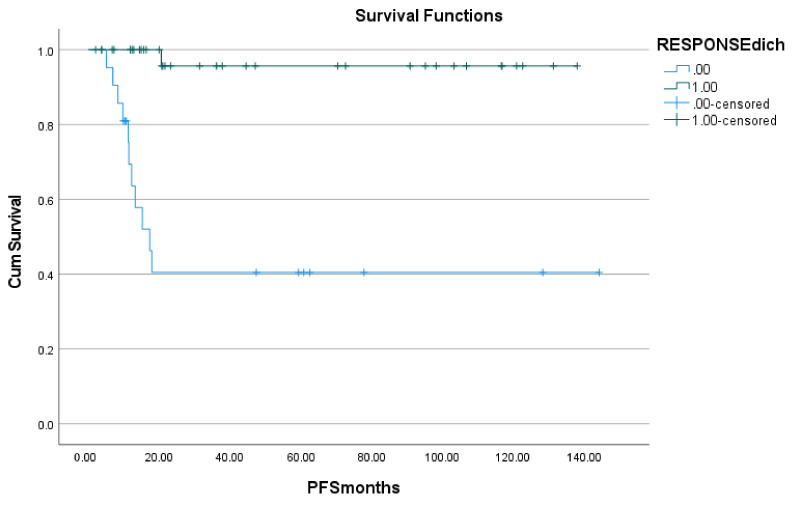
Kaplan-Meier plot of progression-free survival (PFS) in months as a function of dichotomized treatment response.

**Figure 3:**
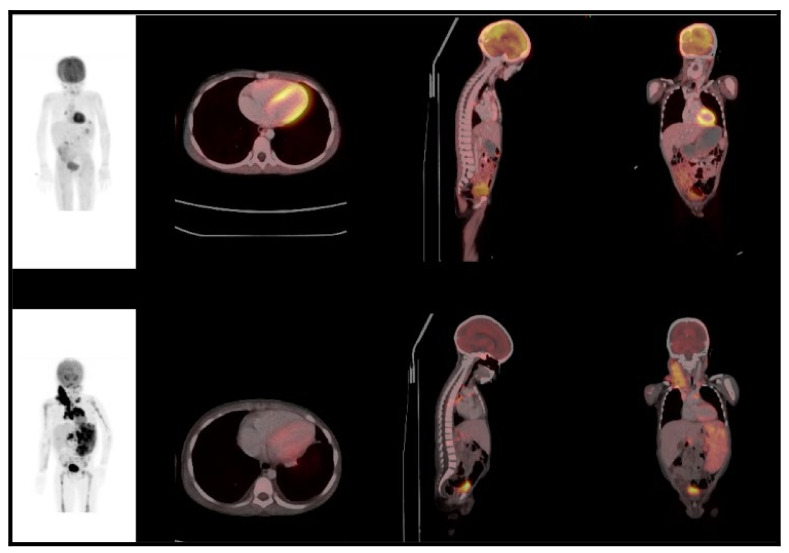
A 6-year-old male with Hodgkin lymphoma. Baseline images (**bottom row**: maximum intensity projection, MIP; axial, coronal, and sagittal fused PET/CT) demonstrated splenic and bone marrow involvement, as well as a pericardial effusion and small bilateral pleural effusions. tMTV-901.83 cm^3^; TLG-3655.06; SUVmax 10.78. After 6 cycles of ABVD (adriamycin, bleomycin, vinblastine, dacarbazine) chemotherapy, a partial metabolic response was noted on the end of treatment PET/CT (**top tow***).* The patient later progressed with a PFS of 11.2 months.

At the time of data analysis, 19 patients had progressed. The median progression-free survival in the patients included in the study was 20.0 months (range 0.3–148 months). On univariate analysis, the presence of bone marrow involvement (*p* = 0.028), the presence of pleural effusion (*p* < 0.001), Hb-level (*p* = 0.039), HIV-status (*p* = 0.036), and treatment response (*p* < 0.001) were predictive of progression-free survival (see Table 2). When included in the multivariate model together with age and gender as covariates, only treatment response (*p* = 0.029) and bone marrow involvement (*p* = 0.043) retained their prognostic value (see Table 3, Figure 2, and Figure 3).

### 3.4. Overall Survival

**Figure 4:**
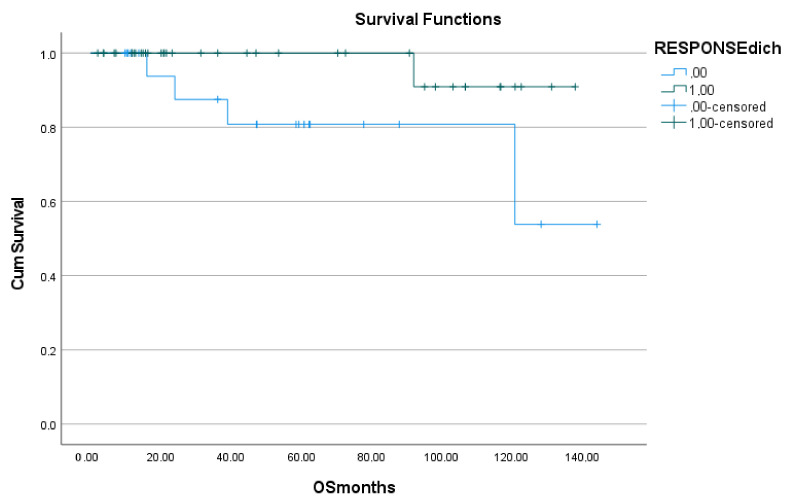
Kaplan-Meier plot of overall survival (OS) in months, as a function of dichotomized treatment response.

At the time of data analysis, 12 patients had died, and all deaths were directly related to the patients’ underlying diseases. On univariate analysis, the presence of pleural effusion (*p* = 0.017) and treatment response (*p* = 0.05) were predictive of overall survival (see Table 2). When included in the multivariate model, together with age and gender as covariates, only treatment response retained its prognostic value (*P* = 0.05) (see Table 3, Figure 4).

## 4. Discussion

Our study aimed to evaluate the prognostic value of tMTV and TLG (compared with SUV_max_) on baseline F-18 FDG PET/CT imaging in predicting metabolic response to treatment, overall survival, and progression-free survival in paediatric Hodgkin lymphoma. Additionally, the utility of other clinical, biochemical, and image-derived risk factors, as well as response to treatment on EOT PET (as defined by the Deauville criteria), were assessed as predictors of outcome.

A multitude of previous studies have demonstrated that response assessment using F-18 FDG PET/CT significantly correlates with patient outcome in both limited-stage and advanced-stage Hodgkin lymphoma and is more accurate than conventional imaging [14,15]. In limited stage disease, PET evaluation at the end of treatment is highly predictive of progression-free survival (PFS) and overall survival (OS), with a negative PET scan conferring an excellent prognosis, whereas in advanced stage disease, consolidation radiation therapy may be omitted in PET-negative patients after completion of chemotherapy (even with residual masses on CT) [3]. On the other hand, PET-positive patients have a significantly increased risk of recurrent or progressive disease, and the findings of residual metabolic activity can direct further therapy [3,4]. In a study of 72 children with Hodgkin lymphoma, it was found that the Deauville criteria were superior to other methods in the prediction of outcome using interim PET data [16]. Similarly, the value of F-18 FDG PET/CT at the end of therapy was demonstrated in the present study, in that treatment response (as evaluated using the Deauville criteria on EOT PET) was significantly related to both PFS and OS on both univariate and multivariate analyses.

In contrast, none of the metabolic parameters in question (SUV_max_, MTV, and TLG) featured as predictors for either PFS or OS, and only MTV was predictive of treatment response. Although the prognostic value of SUV_max_ has been demonstrated in multiple studies, its reliability may be compromised by a host of factors, including but not limited to blood glucose levels, body weight, administered FDG dose (including the effects of extravasation), residual activity left in the syringe, decay of injected dose, uptake time, the presence of partial volume effects, and reconstruction parameters [2,6,7,8,9]. In addition, SUV_max_ is only measured by the highest image pixel in the tumor region and therefore does not show the metabolic activity of the entire tumor [17]. These inherent variations are the likely reasons that our study failed to demonstrate SUV_max_ as a predictor of outcome.

On the other hand, failure to demonstrate the prognostic value of MTV and TLG was somewhat unexpected, given that these metabolic parameters allow for measurement of the whole-body tumor burden [10,11,12]. MTV is the sum of voxels with an increased SUV inside the tumor lesion and represents the volume of viable tumor cells (the tumor volume with increased metabolism) [6,17]. TLG is the product of the MTV and SUV_mean_ of the lesion and therefore reflects tumor metabolic activity and volume [8]. It follows that tMTV or WB-MTV (total or whole-body MTV) is the sum of the metabolic volumes of all lesions, whereas WB-TLG is the sum of all lesion TLGs. Current literature suggests that these parameters may confer great prognostic value when measured on baseline F-18 FDG PET/CT; however, there are limited studies evaluating their performance, particularly in paediatric lymphoma [2]. Furthermore, data are somewhat conflicting: in a recent study by Zhou et al., it was found that the TLG of baseline F-18 FDG PET/CT was the only independent prognostic factor for PFS in paediatric lymphoma, with higher levels predicting significantly lower PFS than lower levels. The tMTV and TLG were both associated with OS and were deemed to be more reliable indicators of treatment response when compared to SUV_max_, which failed to demonstrate a statistically significant difference between progression and progression-free groups [2]. However, tMTV was not found to be an independent prognostic factor for PFS [2]. This contrasts with the findings of Yang et al. and Mathew et al., who recently demonstrated the utility of baseline tMTV as a prognostic marker in paediatric lymphoblastic lymphoma and paediatric anaplastic large cell lymphoma, respectively [6,8]. On the other hand, Chen et al. found that baseline tMTV as well as TLG are both strong independent prognostic factors for paediatric NHL, outperforming other clinical-pathological risk factors (including serum LDH and bone marrow involvement on biopsy) in predicting survival [10]. This inconsistency (which exists across multiple studies) may be attributed to the lack of a standardized method for measuring tMTV [2]. In many studies, tMTV is measured using a threshold method based on 25–50% of the SUV_max_, usually 41% as recommended by the EANM [10,18]. Meignan et al. found the fixed 41% threshold to be reproducible and provided the best concordance between measured and actual volumes [19]. Zhou et al. used a fixed SUV_max_ threshold of 2.5 to determine the MTV [2]. Another issue is the physiological distribution of FDG in organs that may be affected by lymphoma, such as the liver, spleen, thymus, and reactive marrow. All these factors highlight the importance of developing an accurate and well-standardized method to define tumor volume, which is currently not well established, especially in the context of paediatric lymphoma where measurement of tumor volumes is more complex. It is also worth noting that TLG derives from SUV; hence all the limitations of SUV may be reflected in TLG.

Other important considerations that may account for the discrepancies is the heterogeneity of the patients represented by the different studies (i.e., varying histologies) and the different histologies within the same study. For example, in the study by Zhou et al., the patient cohort comprised 16 patients with Hodgkin lymphoma and 31 patients with non-Hodgkin lymphoma. The present study represents a relatively large cohort of patients (*n* = 69), all with the same histological type (Hodgkin lymphoma). To our knowledge, this is the largest study to date in the evaluation of MTV and TLG as prognostic markers in paediatric patients with solely Hodgkin lymphoma.

Interestingly, we did find that MTV was predictive of treatment response. Similar findings were reported by Rogasch et al. [20]. In this retrospective analysis of 50 children with HL, consecutively treated according to the EuroNet-PHL-C1 or –C2 treatment protocol, it was found that a high total MTV on pre-therapeutic F-18 FDG PET/CT was the best predictor of inadequate response to induction therapy of all pre-therapeutic FDG PET parameters (including SUV and TLG) [20]. This finding was consistent across both low and high stages, as well as for the three different treatment groups [20]. The concurrence in findings may be reflective of similar study populations, in terms of histology (i.e., only patients with Hodgkin lymphoma).

Although an in-depth analysis of the findings of metabolic parameters as prognostic markers in adult lymphoma is beyond the scope of this study, it is worth mentioning that tMTV and TLG have been found to be significant prognostic factors in adult patients with various histological sub-types of lymphoma [10]. As with the findings in paediatric lymphoma, discrepancies exist regarding the precise utility of MTV versus TLG, with some studies only supporting one of the parameters as a prognostic marker while others do not clearly define a superior parameter. A very important consideration that is unique to paediatric patients is the measured whole-body tumor burden (tMTV and TLG) relative to body size. Indeed, the comparison of a certain numerical tumor volume in a 15 kg 4-year-old to that of a 55 kg 18-year-old seems preposterous, yet no paediatric study to date (including the present study) has taken this inherent flaw into account. This may be a significant factor in the inconsistent findings across studies. Furthermore, the number of studies and size of patient cohorts are generally much larger in the published data for adult lymphoma, highlighting the need for further research in paediatric lymphoma.

Our finding that the presence of effusion on baseline PET imaging is predictive of OS and PFS is concordant with the conclusions of Zhou et al. [2]. On the other hand, Zhou et al. and Chen et al. did not find prognostic value in bone marrow involvement, which contrasts with our finding that bone marrow involvement on baseline PET is predictive of PFS on both univariate and multivariate analyses [2,10]. Furthermore, Chen et al. noted that bone marrow involvement identified only by PET demonstrated inferior prognostic value, when compared with bone marrow biopsy [10]. A likely reason for this discrepancy is that our study only considered patients with Hodgkin lymphoma, whereas Chen et al. looked at patients with mature B-cell lymphoma and Zhou et al. had a heterogeneous patient cohort (34% Hodgkin lymphoma and 66% various sub-types of non-Hodgkin lymphoma). The value of FDG PET/CT in defining bone marrow involvement in Hodgkin lymphoma is well established in the literature. In the case of positive findings, i.e., focal or diffuse heterogeneous (multifocal) uptake, there is emerging evidence that the need for bone marrow biopsy may be obviated [1,3]. Therefore, our study highlights the prognostic value of bone marrow involvement (identified on baseline PET) in Hodgkin lymphoma.

In this study, we also aimed to investigate the impact of HIV on treatment response and survival. It is known that HIV-positive children are at higher risk of developing lymphoma, as HIV itself causes immunologic and cellular changes that increase susceptibility to other viruses and enhance their oncogenic potential, irrespective of anti-retroviral (ART) administration and CD4+ count [21]. Furthermore, it has been noted that even with early diagnosis and early initiation of ART (before advanced immunosuppression develops), concurrent HIV serves as an additional diagnostic and therapeutic challenge in childhood lymphoma [22]. Our study demonstrated that HIV status is predictive of PFS, but not of OS. The current literature evaluating the impact of HIV on metabolic tumor burden, treatment outcome, and survival in paediatric lymphoma is sparse; however, in a study by Lawal et al. on the role of F-18 FDG PET/CT in evaluating the impact of HIV infection on tumor burden and therapy outcome in adult patients with Hodgkin lymphoma, it was shown that HIV is the only significant predictor of poor therapy outcome. HIV was not, however, associated with a higher tumor burden, and neither SUV_max_ nor MTV nor TLG was a predictor of poor outcome. The impact on survival was not, however, investigated in this study [23]. In another study, Lawal et al. also demonstrated that there was no significant difference in FDG metabolic parameters (MTV, TLG, SUV_max_, and SUV_mean_) between HIV-infected and uninfected adult patients with Hodgkin lymphoma [24]. Of note is that Hodgkin lymphoma is regarded as an HIV-defining malignancy in adults, but the same trend has not yet been confirmed in children in African studies [5]. It is evident that further research is needed in this area.

The strengths of the present study are reflected in the fairly large sample size, the significant follow-up period, and the homogeneity in terms of histological sub-type, when compared with similar studies. This is important, as Hodgkin lymphoma and non-Hodgkin lymphoma are biologically and prognostically different. Furthermore, end-of-therapy PET parameters were always measured after first-line therapy (i.e., approximately 6 weeks after completion of 6 cycles of ABVD) for standardization purposes; if the patient went on to receive second-line chemotherapy or radiation therapy, any subsequent PET scans would only be considered for determining progression status (not for EOT PET response assessment). If the patient cohort was heterogeneous and included those with non-Hodgkin lymphoma, this would not have been possible due to varying treatment protocols.

In spite of the above, we recognize several limitations. First, the inherent limitations of a single-center retrospective study are noted. However, as Steve Biko Academic Hospital is a major central academic hospital, specialized departments such as Nuclear Medicine and Paediatric Oncology serve a huge patient base spanning multiple provinces, including Gauteng, Limpopo, and Mpumalanga.

Second, histological confirmation of disease noted as recurrence on PET was not always confirmed, giving rise to the possibility of false positives due to infectious or inflammatory causes. However, in reality, this was infrequently a concern, as no findings were interpreted in isolation; the full clinical and biochemical picture, as well as follow-up imaging, was used to determine presence of recurrence when histology was not available.

Third, in some cases, the EOT PET was obtained at 2 weeks following chemotherapy completion, resulting in concern about false positive findings, as it is recommended that at least 3 weeks (and ideally 6 weeks) should elapse prior to obtaining EOT PET. However, in the majority of cases (all except two) in which EOT PET was obtained at 2 weeks post-therapy completion, a complete metabolic response was noted; therefore, this was unlikely to have affected the results in any significant way.

As alluded to previously, the consideration of MTV and TLG in proportion to the size of the paediatric patient was not accounted for. In future studies, this may be an area of great interest to explore. Retrospective analysis of data obtained from larger patient cohorts (such as data from the EuroNet-PHL-C1 and C2 trials, which aim to reduce unnecessary treatment for children and young adults with classical Hodgkin lymphoma, thus reducing late effects from radiation therapy) may be of great value, considering the standardization of follow-up and treatment regimes in these trials, limiting confounding factors [25,26,27]. Alternative methods for response assessment, such as quantitative measures of FDG uptake in residual mass (quantitative PET [qPET] method) may also be investigated, and the qPET values may easily be translated into Deauville scores [1,11,12]. Evaluation of therapeutic response assessment of lymphoma using the PERCIST criteria (PET response criteria in solid tumors) may also be explored in future research.

There is also a need to establish the value of F-18 FDG PET/CT in response evaluation of paediatric non-Hodgkin lymphoma, which (in contrast to Hodgkin lymphoma) is not well-defined [3,8,10,11,12]. Overall, there is a need to improve the response criteria to increase the prognostic value of PET in non-Hodgkin lymphoma.

Finally, an exciting consideration for further research would be investigation of the utility of F-18 FDG PET/MRI (magnetic resonance imaging) versus F-18 FDG PET/CT in the management of patients with paediatric lymphoma. Compared to CT, MR imaging has the advantage of superior soft tissue resolution and functional imaging capabilities, while offering reduced radiation exposure. This is of great relevance for paediatric patients, especially those who will require multiple scans during follow-up, as is the case in paediatric lymphoma [3]. Although sensitivity of whole-body diffusion-weighted MR for lesion detection is reported at 96%, a major weakness of MR imaging is its low specificity for evaluation of post-therapeutic changes due to persisting bone marrow edema, necrotic tissue, and contrast enhancement in successfully treated lesions [3]. Thus, a combined approach of PET/MR incorporating metabolic information may enhance available diagnostic capabilities. In a prospective study by Sher et al., comparing the diagnostic performance of PET/MRI versus PET/CT in lesion detection, lesion classification, and disease staging in paediatric lymphoma, it was found that the two modalities are comparable, with the advantage of reduced radiation exposure of PET/MRI [28]. These findings were confirmed by Verhagen et al. in a recent prospective study on the use of PET/MRI for staging and interim response assessment in paediatric and adolescent Hodgkin lymphoma, using FDG PET/CT as the reference standard [29]. Although FDG PET/MRI is clearly a promising alternative for staging and response assessment, it should be kept in mind that sample sizes in the abovementioned studies were small (24 and 25 patients, respectively). Furthermore, limitations such as time for image acquisition (and the need for sedation) and costs/reimbursement for MRI imaging need to be considered. Overall, further research is needed in this area to define the role of PET/MRI.

The findings of the present study should not serve in any way to under-represent the immense value of F-18 FDG PET/CT in paediatric lymphoma. If anything, it is encouraging that even patients with very large whole-body tumor burden may still achieve a complete metabolic response to therapy with subsequent good outcomes. Measuring MTV and TLG is time-consuming, and therefore the true utility of these parameters needs to be established in future studies before incorporating them into routine clinical practice.

## 5. Conclusions

The presence of bone marrow involvement and effusion on baseline F-18 FDG PET/CT and treatment response on end-of-treatment F-18 FDG PET/CT proved to be significantly related to PFS, whereas only effusion and treatment response proved to be significantly related to OS. Neither MTV nor TLG nor SUV_max_ was predictive of OS or PFS, whereas only MTV predicted treatment response.

Regarding clinical parameters, only HIV status and baseline haemoglobin were predictive of PFS. This highlights the value of EOT F-18 FDG PET/CT in prognostication in paediatric and adolescent Hodgkin lymphoma. Further studies are required to define the true significance of metabolic parameters on baseline F-18 FDG PET/CT in paediatric lymphoma.

## Figures and Tables

**Table 1:** Patient characteristics.

Age (Median/Range)	11 Years (4–20 Years)
4–8 years	30 (43%)
9–12 years	19 (28%)
13–15 years	11 (16%)
16–18 years	5 (7%)
19–20 years	4 (6%)
Gender (F/M)	33/36 (48/52%)
Sub-type	
Nodular sclerosing	31 (45%)
Mixed cellularity	16 (23%)
Lymphocyte rich	5 (7%)
Lymphocyte depleted	2 (3%)
Not specified	15 (22%)
Disease stage	
Stage I	1 (1%)
Stage II	25 (36%)
Stage III	17 (25%)
Stage IV	26 (38%)
B-symptoms	
Present	28 (41%)
Absent	30 (43%)
Unknown	11 (16%)
HIV status	
Positive	13 (19%)
Negative	51 (74%)
Unknown	5 (7%)
Hb	
Reduced (<10.5 g/dL)	28 (41%)
Normal (≥10.5 g/dL)	34 (49%)
Unknown	7 (10%)
Albumin	
Reduced (<35 g/L)	34 (49%)
Normal (≥35 g/L)	25 (36%)
Unknown	10 (15%)
LDH	
Elevated (>2× ULN)	14 (20%)
Normal (≤2× ULN)	16 (23%)
Unknown	39 (57%)
Bone marrow involvement	
Present	20 (29%)
Absent	49 (71%)
Splenic involvement	
Present	32 (46%)
Absent	37 (54%)
Liver involvement	
Present	6 (9%)
Absent	63 (91%)
Lung involvement	
Present	9 (13%)
Absent	60 (87%)
Pleural effusion	
Present	9 (13%)
Absent	60 (87%)
Bulky disease	
Present	2 (3%)
Absent	67 (97%)

F = female, M = male, HIV = human immunodeficiency virus, Hb = haemoglobin, LDH = lactate dehydrogenase.

**Table 2:** Univariate analysis of the relationship between the studied variables and progression-free and overall survival. Statistically significant values are highlighted in bold.

	Progression-Free Survival *p* (Log-Rank)	Overall Survival *p* (Log-Rank)
Age	0.117 (NS)	0.111 (NS)
Gender	0.556 (NS)	0.975 (NS)
Disease stage	0.346 (NS)	0.586 (NS)
B-symptoms	0.083 (NS)	0.504 (NS)
HIV-status	**0.036**	0.059 (NS)
Hb levels	**0.039**	0.350 (NS)
Albumin levels	0.398 (NS)	0.323 (NS)
LDH levels	0.147 (NS)	0.203 (NS)
Bone marrow involvement	**0.028**	0.268 (NS)
Spleen involvement	0.479 (NS)	0.907 (NS)
Liver involvement	0.539 (NS)	0.958 (NS)
Lung involvement	0.675 (NS)	0.753 (NS)
Pleural effusion	**<0.001**	**0.017**
Bulky disease	0.0670 (NS)	0.546 (NS)
MTV	0.065 (NS)	0.263 (NS)
TLG	0.099 (NS)	0.308 (NS)
SUVmax	0.379 (NS)	0.799 (NS)
Treatment response	**<0.001**	**0.050**

NS = not significant, Hb = haemoglobin, LDH = lactate dehydrogenase, HIV = human immunodeficiency virus, MTV = metabolic tumor volume, TLG = total lesion glycolysis, and SUVmax = maximum standardized uptake value of the most active lesion. Statistically significant values are highlighted in bold.

**Table 3:** Multivariate analysis of the relationship between the significant variables derived from the univariate analysis and progression-free and overall survival.

	Progression-Free Survival *p* (Log-Rank)	Overall Survival *p* (Log-Rank)
Bone marrow involvement	**0.043**	-
Treatment response	**0.029**	**0.05**

Statistically significant values are highlighted in bold.

## Data Availability

The data presented in this study are available on request from the corresponding author. The data are not publicly available due to patient confidentiality.
